# Fundamental motor skills, executive functions, and academic achievement of Chinese schoolchildren: Testing a mediating model

**DOI:** 10.1371/journal.pone.0336470

**Published:** 2025-11-21

**Authors:** Lijing Wang, Jiancui Sun, Lijuan Wang, Danheng Zheng, Yulan Zhou

**Affiliations:** 1 School of Physical Education, Minnan Normal University, Zhangzhou, China; 2 College of Sports Medicine and Rehabilitation, Shandong First Medical University, Taian, China; 3 School of Physical Education, Shanghai University of Sport, Shanghai, China; 4 College of Physical Education and Health Sciences, Zhejiang Normal University, Jinhua, China; Mae Fah Luang University School of Anti Aging and Regenerative Medicine, THAILAND

## Abstract

**Background:**

Researchers have observed the relationship between children’s fundamental motor skills (FMS) and their academic achievement. However, the inner workings of this correlation remain underexplored, especially in the context of China. This study addresses this gap by investigating the associations among FMS, such as locomotor, object control, and stability skills, executive function (EF), such as inhibition control and working memory, and academic achievement among Chinese schoolchildren. Furthermore, this research determines how EF mediates the relationship between FMS and academic achievement and whether gender and age moderate the mediating process.

**Method:**

This research involved 733 primary schoolchildren aged 6–12 years from China. First, the Test of Gross Motor Development-Third Edition and the Körperkoordinationstest Für Kinder were used to measure FMS. Then, the Childhood Executive Functioning Inventory was employed to evaluate EF, while standardized written examinations in Chinese and mathematics subjects were used to determine academic achievement.

**Results:**

Regression analyses were conducted. Results revealed that the children’s performance in Chinese was negatively correlated with their working memory (*β *= −0.129, *p *= 0.005) and inhibition control (*β* = −0.191, *p *< 0.001) but positively linked to object control (*β* = 0.198, *p* < 0.001). Meanwhile, children’s performance in mathematics had a negative relationship with inhibition (*β* = −0.246, *p *< 0.001) but positive relationships with object control (*β* = 0.095, *p* = 0.009) and stability skills (*β *= 0.096, *p *= 0.010). In addition, a mediation analysis was conducted. Results revealed that EF had a partially mediating effect on the relationship between FMS and academic achievement in Chinese (*β* = 0.110, 95% *CI* [0.051, 0.175]) and mathematics (*β* = 0.134, 95% *CI* [0.070, 0.189]). In the mathematics model, it is only found that age significantly and negatively moderates the path between EF and mathematics (*β* = −0.627, 95% *CI* [−0.698, −0.332], *p* < 0.001).

**Conclusions:**

This study highlights the critical role of FMS and EF in Chinese schoolchildren’s academic achievement. The outcomes of this work suggest that educators can bolster FMS and EF through well-crafted, engaging, and encouraging interventions or programs. In turn, these designs can support the academic achievement of schoolchildren. Strategies aimed at improving object control can foster children’s performance in Chinese and mathematics. Meanwhile, programs focused on developing stability skills can be implemented to enhance performance in mathematics, especially for younger primary schoolchildren.

## Introduction

Academic achievement refers to the degree to which students accomplish their educational objectives [1]. High levels of academic achievement are linked to individuals’ successful participation in daily activities and social interactions and may influence the development of children [2]. By contrast, low levels of academic achievement are linked to health issues, such as mental illness, including anxiety and depression, and weak physical well-being [3]. The academic success of children is shaped by a combination of various factors, such as the personal, social, and institutional circumstances of the children [3]. Given this context, the effects of fundamental motor skills (FMS) and executive function (EF) on academic achievement have attracted growing interest [4–7].

Piaget’s cognitive development theory suggests that cognition is an individual’s personal actions and interactions with the world [8]. The theory proposes that children progress through four distinct stages: the Sensorimotor Stage (birth to 2 years), the Preoperational Stage (2–7 years), the Concrete Operational Stage (7–11 years), and the Formal Operational Stage (11 years and beyond). It is important to note that the age ranges associated with these stages are not absolute; individual differences exist and cultural factors may also exert influence on the developmental trajectory through these stages. Researchers have been guided by this theory in exploring the link between children’s FMS and academic performance [9–11] Building on this research, the present study examines this correlation further to offer significant insights. FMS refers to the essential elements for executing intricate and advanced movements in various activities, including sports, games, and specific physical tasks [12]. These skills are typically acquired during an individual’s early years. They consist of locomotor skills (e.g., running, hopping, and sliding), object control skills (e.g., dribbling, throwing, catching, and kicking), and stability skills (e.g., swinging, turning, and balancing on one foot) [13]. FMS helps children to become capable and self-sufficient in performing their daily tasks. Moreover, it forms the basis of children’s physical, psychological, and social development and active lifestyle [14,15].

Some researchers have examined children’s learning performance in reading and mathematics and found that FMS are positively correlated with academic performance to a small to strong extent [9,16–20]. For example, de Bruijn et al. (2019) [9] reported that FMS was positively correlated with children’s reading and mathematics scores; however, no such relationship exists in children’s performance in spelling. Meanwhile, Son and Meisels (2006) [16] conducted a longitudinal study and determined that the early development of FMS among schoolchildren can promote achievements in reading and mathematics. Studies have also identified balancing as the skill with the positive correlation with academic performance. For instance, Lonnemann (2011) [17] demonstrated the positive link between balancing and arithmetic abilities among children aged 8–10 years in Germany. Meanwhile, Lima et al. (2020) [18] observed first- to fifth-grade children in Australia for one year and concluded that balancing skills had a positive relationship with Danish language and mathematics whereas precision throw did not. Additionally, Haapala et al. (2014) [19] conducted a longitudinal study of first- to third-grade children’s motor and academic skills. The researchers concluded that poor balance is related to difficulties in reading comprehension with a stronger effect on boys than on girls.

Research has also shown that cognitive abilities, including EF, can influence academic outcomes, especially reading, mathematics, and spelling [21–23]. EF refers to advanced cognitive processes that support adaptable and goal-oriented behavior [24]. The two most recognized aspects of EF are inhibition control, which is the ability to overcome temptations and impulses, and working memory, which is the capacity to hold and yield information creatively. In addition, researchers have recognized cognitive flexibility as another important aspect. This concept pertains to modifying one’s ideas and approaches to meet changing circumstances, such as demands, rules, and priorities [25,26]. Cross-sectional studies have confirmed a strong connection between children’s EF and academic achievements in various subjects, including reading, mathematics, and spelling [21,27,28]. For instance, Cortes Pascual et al. (2019) [29] conducted a literature review and meta-analysis of 19 studies, which revealed that improving children’s EF can enhance their academic performance (language and mathematics). They also observed that working memory is the strongest determinant of a child’s positive learning outcomes among all EF components.

Although a direct relationship has been established between FMS and academic achievement, this complex correlation may be mediated by specific factors (e.g., EF) [30–37]. Rigoli et al. (2012) [32] found that motor coordination has an indirect connection with academic achievement (reading, spelling, and mathematics) via working memory in typically developing adolescents aged 12–16 years. In addition, Schmidt et al. (2017) [33] indicated that three motor abilities, namely, cardiovascular fitness, muscle strength, and coordination, had a positive correlation with academic achievement of reading, spelling, and mathematics of children. Moreover, EF fully mediated only the relationship between motor coordination and academic achievement. Cadoret et al., (2018) [34] found that cognitive ability such as working memory mediated the relationship between the FMS and early academic achievement (reading and mathematics) in children. Murrihy et al. (2017) [36] confirmed that in children aged 8–12, short-term memory fully mediated the relationship between psychomotor ability and reading and math achievement.

China’s unique cultural and educational system may shape the dynamics among children’s FMS, EF, and academic achievement in ways that differ from the trends in Western countries. For instance, China is deeply rooted in Confucian culture, which gives a high value to educational excellence. Hence, parents and teachers closely monitor children’s learning experiences and academic performance [38]. In relation to this focus on children’s progress, EF is believed to have a strong influence on academic performance [5]. Even at an early age, children are taught to hone EF skills, such as sitting still and listening attentively in class [39]. As such, research has revealed that Chinese children develop stronger EF skills than some Western children [40–42]. Given the emphasis on these skills, Chinese teachers and parents have overlooked the physical activity and FMS of children. They view these skills as time consuming and unnecessary, that is, a significant portion of time spent in developing these skills can be utilized in academic learning instead [43,44]. The FMS, EF, and academic performance of children can have varying degrees of importance in specific contexts of different cultures and countries. However, extensive research is needed to determine the correlation among FMS, EF, and academic achievement among children in China.

### Present study

**Fig 1:**
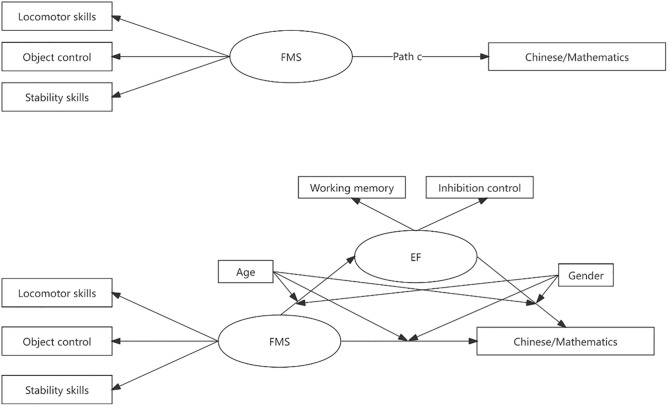
Hypothetical mediation model. The influence of FMS (independent variable) on academic achievement (dependent variable) through EF (mediator). Path a, the association between FMS and EF; Path b, the association between EF and Chinese/mathematics achievement; Path c, the total effect of FMS on Chinese/mathematics achievement; Path c’, the direct effect of FMS on Chinese/mathematics achievement.

This study addresses research gaps by exploring the association among the FMS (locomotor, object control, and stability skills), EF (inhibition control and working memory), and academic achievement (Chinese and mathematics) of primary schoolchildren in China. It further investigates the mediation of EF in the relationship between FMS and academic achievement and the moderating effects of age and gender variables in the mediating role. Research has indicated that the relationship among these variables varies depending on the subjects [4,9,29,31]. For example, Aadland (2017) [4] examined 697 fifth-grade students and found that boys’ FMS correlated positively with mathematics but negatively with English, while girls had positive correlations with all academic indicators. A study by de Bruijn (2019) [9] on 891 third-and fourth-grade primary school students showed that the FMS was significantly correlated with reading and math scores, but had no association with spelling scores. The researchers speculated that this difference may be related to the different brain regions and neural networks recruited and activated during the skill acquisition process. Chinese and mathematics form the core of the primary school curriculum in China. Knowns as the “Two Basics”, these two subjects are the cornerstone of primary learning and are prioritized with the greatest amount of instructional time and institutional focus [45]. Therefore, the current study focuses on Chinese and mathematics to assess the academic performance of children in primary schools in China. It analyzes the correlation among FMS, EF, and academic achievement in each subject. Then, the following hypotheses are proposed. 1) FMS and EF are strongly associated with children’s academic achievement in Chinese and mathematics. 2) EF mediates the relationship between FMS and academic performance in Chinese and mathematics. 3) The degrees of relationship among FMS, EF, and academic achievement differ in Chinese and mathematics subjects. 4) The variables of age and gender moderated the mediating effect of the model. Fig 1 presents the hypothetical mediation model.

## Method

### Participants

**Fig 2:**
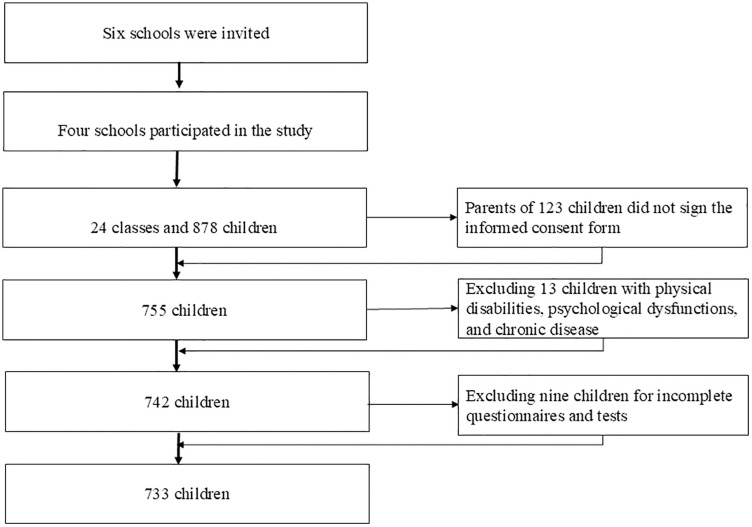
Flow of sample recruitment and screening.

The recruitment of the original sample started on 15 September 2022 and ended on 3 July 2023. Multistage cluster random sampling was used to select participants from six primary schools in two districts with three school in each district of the city of Jinhua, which is located in eastern China. After contacting principals of schools within these districts, four schools consented to participate in the study. The four schools are situated in the downtown, urban, and suburban areas of Jinhua respectively, representing different economic contexts. Two medium-sized schools are located in the city center including one public school with a student-teacher ratio of 1:20.1, and one private school with a ratio of 1:18.5. There is a small private school in the urban area with a student-teacher ratio of 1:16, while the suburban district has a small public school with a ratio of 1:15. The four primary schools have six grades with children ranging from 6 to 12 years old. One class was randomly selected from each grade from grade 1–6 and all students in each class were invited to participate, resulting in a sample of 24 classes and 878 children. Then, consent forms were disseminated to the children and their parents or guardians; 755 children opted to participate, thus yielding an 86% response consent rate. However, 13 children were omitted from the sample because of the exclusion criteria. Three children had a physical disability, two had psychological dysfunction, and eight had chronic illnesses. These conditions entailed that the excluded children could not execute normal physical tasks and academic learning. Furthermore, the preliminary examination of the raw data revealed that nine participants failed to finish the FMS tests because of illness or missing responses to the EF questionnaires. These participants also had to be removed, thus resulting in a final sample of 733 children. Fig 2 presents the sample recruitment process. The children were from 6 to 12 years old (*M* = 8.74, *SD* = 1.69), and 349 (47.6%) were females. Table 1 shows the descriptive statistics of the samples.

**Table 1:** Descriptive statistics (N = 733).

	Total
Grade (n(%))	
1	122 (16.6%)
2	132 (18.0%)
3	126 (17.2%)
4	162 (22.1%)
5	127 (17.3%)
6	64 (8.8%)
Mother’s educational (n(%))	122 (16.6%)
Level 1	132 (18.0%)
Level 2	126 (17.2%)
Level 3	162 (22.1%)
Level 4	127 (17.3%)
Father’s educational (n(%))	64 (8.8%)
Level 1	122 (16.6%)
Level 2	132 (18.0%)
Level 3	126 (17.2%)
Level 4	162 (22.1%)
Average annual household income (n(%))	
< 9000	48 (6.5%)
9000 ~ 30000	98 (13.4%)
30001 ~ 100000	513 (70.0%)
> 100000	74 (10.1%)

Note: Mother’s/Father’s educational level: 1, junior high school and below; 2, High school or secondary vocational;

3, junior college; 4, Bachelor’s degree or above

### Measures

#### FMS.

Locomotor and object control skills were assessed using the Test of Gross Motor Development—Third Edition (TGMD-3). This evaluation comprised two subtests: Locomotor Skills (six items: run, gallop, one-legged hop, skip, jump, slide) and Object Control Skills (six items: two-hand strike, one-hand strike, catch, kick, dribble, overhand throw, underhand throw) [46].

Each TGMD-3 item was evaluated against three to five performance criteria. For example, the catch item included:(1) Preparation phase: hands positioned in front of the body with flexed elbows; (2) Arm extension while reaching for the ball upon arrival; (3) Ball caught exclusively with the hands. Participants performed each skill twice. Each performance criterion was scored binarily (0 = incorrect; 1 = correct). The total score per subtest represented the sum of points across both performances. Maximum possible scores were 46 for locomotor skills (derived from 23 criteria × 2 trials) and 54 for object control skills (27 criteria × 2 trials).“

Meanwhile, stability skills were evaluated using the Körperkoordinationstest Für Kinder (KTK). The KTK consists had four subtests: 1) walking backward three times along each of three balance beams with decreasing widths of 6.0, 4.5, and 3.0 cm (a maximum of 8 steps each time). The number of successful steps of a maximum of 24 steps (eight per trial) was counted for each balance beam, maximum scores of 72 points (3*8*3); 2) hopping on one leg over a foam obstacle with heights increasing by 5 cm consecutively. After a successful hop with each foot, the height was increased by adding a square. Three, two or one point(s) were recorded for successful performance on the first, second or third trial, respectively. A maximum of 39 points (ground level + 12 squares) could be scored for each leg, yielding a possible maximum score of 78; 3) two-legged jumping from one side to another for 15 seconds (the sum of the number of correct jumps in two trials); and 4) moving sideways on wooden boards for 20 seconds (the sum of the number of points in 20 seconds in two trials) [47]. The sum of the raw scores from the four subtests formed the original score, which also served as the motor quotient based on age and gender [48,49]. The reliability and validity of the TGMD-3 and KTK in the study were established [50,51].

#### EF.

The EF was assessed using the Childhood Executive Functioning Inventory (CHEXI), which is an instrument used by parents or teachers to evaluate children aged 4–12 [52]. This measurement has 24 items and four dimensions, namely, working memory (nine items, e.g., “Has difficulty remembering lengthy instructions”), planning ability (four items, e.g., “Has difficulty with tasks or activities that involve several steps”), regulation ability (five items, e.g., “Has clear difficulties doing things he/she finds boring”), and inhibition ability (six items, e.g., “Has a tendency to do things without first thinking about what could happen”) [52]. These dimensions are categorized into two factors, namely, working memory (working memory and planning dimensions) and inhibition control (inhibition and regulation dimensions). In the present study, parents or guardians who accomplished the questionnaire scored each item using a five-point Likert scale (1 = definitely not true to 5 = definite true). High scores signal potential EF difficulties and a decreased ability in the corresponding component. Tests validated the split-half reliability and internal consistency of the Chinese version of CHEXI, which were determined to be satisfactory (α = 0.71 ~ 0.89). In this study, parents assessed their children’s executive functions over the past month based on behavioral manifestations related to these functions in daily life.

### Academic achievement

In China, children’s academic performance in various subjects are evaluated at the conclusion of each semester through grade-specific written examinations. As such, this study used the results of the final examinations in two core subjects of primary schools (Chinese and mathematics) to measure their children’s academic achievement. Moreover, Chinese learning was reported to be moderately related to mathematics learning [53]. The test to evaluate performance in Chinese included reading comprehension, vocabulary, grammar, and composition writing. Meanwhile, the test to assess mathematics included different aspects, such as counting, arithmetic, and geometry. The schools supplied the participants’ grades in the most recent semester. The raw scores ranged from 0 to 100 with high scores representing strong performance.

### Covariates

Demographics data included participants’ age, gender, family income, and mother’s education. These factors are potential covariates because of their influence on the independent and dependent variables [4,31,54].

### Procedure

This research adopted a cross-sectional design during the 2022–2023 academic year and was approved by the Review Board at Zhejiang Normal University (#ZSRT2022048). The researchers ensured that the participants were informed about the objective of the study before collecting data. All participants provided written informed consent prior to data collection. For minors in this study, consent forms were signed by their parents/guardians. Over the span of two months, 12 skilled research assistants evaluated the children’s FMS in their physical education classes. Then, the TGMD-3 and the KTK were administered based on the guidelines set by Ulrich (2016) [46] and by Kiphard and Schilling (2007) [47], respectively. The tests were also documented through video. Afterward, the first author and a researcher in the field watched the video to evaluate and rate the children independently. The average scores of two assessors served as the final scores. The CHEXI questionnaire was inserted into the children’s “take-home” folder on the first day so that their parents could accomplish the survey at home. The questionnaire included items regarding the child’s age and grades, gender, and mother’s educational level. The children needed to return the questionnaire the following day. Then, the teachers evaluated the children’s academic performance at the end of the semester through a written examination disseminated by the school district.

### Data analyses

First, we used SPSS 26.0 software to conduct descriptive and correlation statistical analyses on the research variables. Then, a hierarchical regression analysis was conducted to examine the relationship between the three FMS subscales, the two EF subscales, and academic achievement in Chinese and mathematics. The scores from the Chinese and mathematics examinations were examined separately after controlling for the children’s demographic information. Next, structural equation model (SEM) was used to test the mediation of EF in the relationship between FMS and academic achievement and the moderation of gender and age. Data was analyzed by using Amos 26.0 software [55]. Two structural equation models with maximum likelihood estimation were employed for the hypothesized mediation model. This model posits the mediating role of EF in the relationship between FMS and academic performance in Chinese and mathematics. Chi-square statistic χ^2^ value, goodness-of-fit index (GFI), incremental fit index (IFI), comparative fit index (CFI), standardized root square residual (SRMR), and root mean square error of approximation (RMSEA) were used to determine whether the model was effectively suited with the data [56]. To have an acceptable model fit, the values for CFI and IFI needed to be close to or greater than 0.90. Meanwhile, the values of RMSEA and SRMR needed to be 0.06 and 0.08, respectively, or lower [56]. In addition, the bootstrapping method was used to identify the mediating and moderating relationships between variables. The percentile-based method was used to calculate the confidence intervals, and the sample size was set to 5000. Meanwhile, an interaction term between the independent variable and the moderating variable was constructed, and the influence of the moderating variable on the relationship between the independent variable and the dependent variable was tested.

## Results

Table 2 presents the descriptive statistics and correlation coefficients of the study variables. Object control and stability skills were significantly and positively correlated with academic achievement in Chinese (OC: *r* = 0.271; SS: *r* = 0.200, *p* < 0.01) and mathematics (OC: *r *= 0.182; SS: *r =* 0.197, *p* < 0.01). Meanwhile, locomotor skills had a significantly positive correlation with academic achievement in Chinese (*r *= 0.082, *p* < 0.05) but an insignificant correlation with mathematics (*r *= 0.042, *ns*). All components of FMS had a negative relationship with working memory (OC: *r *= −0.171, *p* < 0.01; SS: *r* = −0.247, *p* < 0.01) and inhibition control (OC: *r* = −0.183, *p* < 0.01; SS: *r* = −0.218, *p *< 0.01) except for the link between locomotor and working memory (*r* = −0.041, *ns*) and inhibition control (*r* = −0. 038, *ns*). In addition, all EF components had a negative correlation with academic achievement in Chinese and mathematics, working memory was significantly negatively correlated with Chinese (*r* = −0.312, *p* < 0.01)) and mathematics scores. (*r* = −0.292, *p* < 0.01), inhibition control was significantly negatively correlated with Chinese (*r *= −0.331, *p* < 0.01) and mathematics scores (*r *= −0.341, *p *< 0.01). There is a moderate correlation between working memory and inhibitory control (*r* = 675, *p* < 0.01).

**Table 2:** Descriptive statistics and Pearson’s correlation for variables (N = 733).

Variable	M ± SD	Locomotor	OC	Stability	WM	Inhibition	Chinese
Locomotor	38.7 ± 6.2	–					
OC	36.1 ± 3.5	−0.005	–				
Stability	89.5 ± 14.0	0.139^**^	0.208^**^	–			
WM	1.56 ± 0.38	−0.041	−0.171^**^	−0.247^**^	α = 0.89		
Inhibition	1.38 ± 0.37	−0.308	−0.183^**^	−0.218^**^	0.675^**^	α = 0.71	
Chinese	78.6 ± 6.0	0.082^*^	0.271^**^	0.200^**^	−0.312^**^	−0.331^**^	–
Mathematics	84.8 ± 3.0	0.042	0.182^**^	0.197^**^	−0.292^**^	−0.341^**^	0.108^**^

Note. M = Mean; SD = Standard deviation; OC: Object control; WM: Working memory; ^*^: *p* < 0.05; ^**^: *p* < 0.01.

Hierarchical regression analyses were used to determine the relationships between FMS components (locomotor, object control, and stability skills) and EF components (inhibition control and working memory) and their correlation with academic achievement in Chinese and mathematics. Model 1 accounted for the demographic variables of age, gender, family income, and mother’s education level. Then, Model 2 incorporated the two components of EF. Model 3 included the three FMS components. Academic performance in Chinese had a negative association with working memory (*β* = −0.129, *p* = 0.005) and inhibition control (*β *= −0.191, *p* < 0.001) but a positive association with object control (*β* = 0.198, *p* < 0.001), thus explaining 17.6% of the variance in the model (Table 3).

**Table 3:** Hierarchical regression in the prediction of Chinese performance (N = 733).

DV	Steps	IV	B	R^2^	β	t	P
Chinese	Model 1			0.001			
		Age	0.031		0.006	0.153	0.878
		Gander	−0.211		−0.018	−0.473	0.637
		Income	−0.099		−0.016	−0.395	0.693
		Education	0.081		0.011	0.274	0.784
	Model 2			0.124**			
		Age	−0.016		−0.003	−0.087	0.931
		Gander	−0.112		−0.009	−0.267	0.789
		Income	−0.084		−0.013	−0.357	0.721
		Education	0.115		0.015	0.412	0.680
		WM	−0.102		−0.161	−3.413	0.001
		Inhibition	−0.144		−0.222	−4.713	0.000
	Model 3			0.176**			
		Age	−0.051		−0.009	−0.278	0.781
		Gander	−0.252		−0.021	−0.618	0.537
		Income	−0.106		−0.017	−0.462	0.644
		Education	0.179		0.024	0.658	0.511
		WM	−0.082		−0.129	−2.790	0.005
		Inhibition	−0.124		−0.191	−4.151	0.000
		OC	0.341		0.198	5.566	0.000
		Stability	0.028		0.064	1.765	0.078
		Locomotor	0.060		0.062	1.813	0.070

Note. DV: Dependent variable; IV: Independent variable. *R*^*2*^ values are cumulative, with each incremental step adding to the variance explained; Both *B* and *β* are used to represent the degree of influence of independent variables on dependent variables. *β* values are standardized regression coefficients at each step of the regression analysis.

Academic performance in mathematics had a negative relationship with inhibition (*β* = −0.246, *p* < 0.001) but had a positive relationship with object control (*β* = 0.095, *p *< 0.01) and stability skills (*β* = 0.096, *p* < 0.05), thus explaining 14.7% of the variance in the model (Table 4).

**Table 4:** Hierarchical regression in the prediction of Mathematics performance (N = 733).

DV	Steps	IV	B	R^2^	β	t	P
Mathematics	Model 1			0.002			
		Age	0.030		0.011	0.297	0.767
		Gander	0.085		0.014	0.379	0.705
		Income	−0.120		−0.038	−0.947	0.344
		Education	0.062		0.017	0.416	0.678
	Model 2			0.125			
		Age	0.003		0.001	0.027	0.979
		Gander	0.136		0.023	0.646	0.519
		Income	−0.116		−0.036	−0.977	0.329
		Education	0.087		0.023	0.621	0.535
		WM	−0.036		−0.112	−2.381	0.018
		Inhibition	−0.087		−0.266	−5.642	0.000
	Model 3			0.147			
		Age	−0.006		−0.002	−0.066	0.947
		Gander	0.093		0.015	0.444	0.657
		Income	−0.130		−0.041	−1.101	0.271
		Education	0.123		0.033	0.886	0.376
		WM	−0.027		−0.085	−1.806	0.071
		Inhibition	−0.080		−0.246	−5.236	0.000
		OC	0.082		0.095	2.626	0.009
		Stability	0.021		0.096	2.588	0.010
		Locomotor	0.008		0.017	0.501	0.617

Note. DV: Dependent variable; IV: Independent variable. *R*^*2*^ values are cumulative, with each incremental step adding to the variance explained; Both *B* and *β* are used to represent the degree of influence of independent variables on dependent variables. *β* values are standardized regression coefficients at each step of the regression analysis.

The hypothesized SEM model in Fig 1 was used to determine how EF mediates the link between FMS and academic achievement in Chinese and mathematics. The two models demonstrated a reasonable fit to the data (Chinese: *χ2/df* = 1.535, *p* = 0.063, *GFI *= 0.992, *IFI *= 0.988, *TLI* = 0.970, *CFI* = 0.987, *RMSEA* = 0.027; mathematics: *χ2/df* = 1.089, *p *= 0.354, *GFI* = 0.994, *IFI* = 0.998, *TLI* = 0.995, *CFI* = 0.998, *RMSEA* = 0.011). Table 5 presents the outcomes of the mediation analysis. FMS had a negative correlation with EF (path a: *β* = −0.476, *p *< 0.001; *β* = −0.452, *p* < 0.001). Meanwhile, EF had a negative relationship with academic achievement in Chinese (path b: *β* = −0.231, *p *< 0.001) and mathematics (path b: *β* = −0.296, *p* < 0.001). Although FMS was positively associated with academic achievement in Chinese (path c: *β *= 0.452, *p* < 0.001) and mathematics (path c: *β *= 0.342, *p *< 0.001), this correlation weakened after including EF into the models (path c’: *β* = 0.342, *p* < 0.001; *β* = 0.208, *p* < 0.01). Nevertheless, the relationship remained significant. Finally, the indirect effects of EF on academic achievement in Chinese and mathematics were statistically significant (*β *= 0.110, 95% *CI* [0.051, 0.175]; *β* = 0.134, 95% *CI* [0.070, 0.189]), thus confirming that EF partially mediated these models. The demographic variables, including age, gender, family income, and mother’s education, were controlled in all models.

**Table 5:** Testing the hypothetical path between FMS and academic performance while controlling for age, gender, family income, and mother’s education level.

DV	Mediator	Total effect(c)	Direct effect(c’)	Path a	Path b	Indirect effect(ab)	Percentile 95% CI		P_M_(%)
							Lower	Upper	
Chinese	EF	0.452^***^	0.342^***^	−0.476^***^	−0.231^***^	0.110	0.051	0.175	23.76
Mathematics	EF	0.342^**^	0.208^**^	−0.452^***^	−0.296^***^	0.134	0.070	0.189	39.88

Note. DV: Dependent variable; IV: Independent variable. CI: confidence interval; ^**^ *p* < 0.01; ^***^ p < 0.001.

Table 6 shows the analysis results of the moderating effect. In the Chinese model, the moderating effects of gender and age on each path are not significant. In the mathematics model, it is only found that age significantly and negatively moderates the path between EF and mathematics (*β* = −0.627, 95% *CI* [−0.698, −0.332], *p* < 0.001).

**Table 6:** The moderating effects of age and gender variables on the mediating path.

Model	IV	DV	Effect	*P*	Percentile 95% CI	
					Lower	Upper
Chinese	Gander*FMS	EF	−0.010	0.875	−0.116	0.096
	Age*FMS	EF	0.035	0.424	−0.051	0.117
	Gander*FMS	Chinese	−0.426	0.211	−0.580	0.229
	Age*FMS	Chinese	0.169	0.432	−0.277	0.544
	Gander*EF	Chinese	−0.031	0.906	−0.251	0.290
	Age*EF	Chinese	0.041	0.856	−0.361	0.291
Mathematics	Gander*FMS	EF	0.037	0.386	−0.048	0.119
	Age*FMS	EF	0.007	0.930	−0.099	0.107
	Gander*FMS	Mathematics	−0.209	0.361	−0.523	0.207
	Age*FMS	Mathematics	0.021	0.827	−0.247	0.300
	Gander*EF	Mathematics	−0.015	0.865	−0.212	0.184
	Age*EF	Mathematics	−0.627	<0.001	−0.698	−0.332

Note. DV: Dependent variable; IV: Independent variable. CI: confidence interval

## Discussion

This study investigated the correlations among EF, FMS, and academic achievements in Chinese and mathematics among schoolchildren in China. FMS and EF were strong predictors of children’s performance in Chinese and mathematics. However, the relationships among the three components of FMS (i.e., locomotor skills, object control, and stability skills), the two components of EF (i.e., working memory and inhibition control), and academic achievement in Chinese and mathematics achievement varied. Furthermore, FMS has an indirect influence on the children’s performance of Chinese and mathematics performance because of the partial mediation of EF.

### Relationship between EF and academic achievement of children

Results showed that EF scores were negatively correlated with children’s academic achievement in Chinese and mathematics. This indicated that children with higher EF performed better in these two subjects because the CHEXI used the reverse scoring. This finding is consistent with previous studies have also reported that enhancing children’s EF can foster increased academic performance. Previous studies have also reported that enhancing children’s EF can foster increased academic performance [21,27,28], especially working memory and inhibitory control. Inhibitory control and working memory represent the two core subcomponents of executive function. While operationally distinct, these capacities exhibit high coordination. Inhibitory control suppresses irrelevant information and impulsive behaviors (e.g., filtering distractions), whereas working memory facilitates temporary information storage and manipulation (e.g., retaining intermediate values during mental arithmetic). Their synergy manifests in problem-solving scenarios: working memory maintains task-relevant data (such as mathematical formulas), while inhibitory control eliminates competing solutions. However, this study demonstrates that the effect of two EF components on children’s scores in Chinese and mathematics differed. Inhibition control was significantly related to academic performance in Chinese and mathematics, whereas working memory only had a significant relationship with academic performance in Chinese. Moreover, inhibition control has a stronger relationship with academic achievement in Chinese (*β *= −0.191, *p* < 0.001) and mathematics (*β* = −0.246, *p* < 0.001) than working memory (*β* = −0.129, *p *< 0.01; *β* = −0.085, *p* > 0.05). By contrast, Cortes Pascual et al. (2019) [29] reported that working memory is the strongest predictor of academic performance among all the components of EF. Chinese classes include lessons on phonology, vocabulary, grammar, semantics, script, and rhetoric. Reading and memorizing Chinese characters, paragraphs, and short articles are the major objectives of the subject. Therefore, working memory can improve children’s performance in Chinese because this ability allows children to retain, store, and process relevant information effectively. Meanwhile, mathematics classes include learning concepts and skills related to numbers, shapes, and measurements as well as their applications. Logical reasoning and arithmetic proficiency are essential for excelling in mathematics [57]. Although working memory can help children perform and remember various mathematical operations and calculations, this skill did not have a significant effect on children’s academic performance in mathematics in this study [58,59]. By comparison, inhibition control had a significant influence on children’s performance in Chinese and mathematics. Stable focus allows children to participate in learning tasks [60,61]. Examinations at the elementary level are not difficult. Therefore, being attentive to an activity and avoiding distractions are key factors to achieving high test scores.

### Relationship between FMS components and academic achievement

FMS was a composite measure of locomotor skills, object control, and stability skills. In this study, such skills had a positive relationship with children’s performance in Chinese and mathematics. This outcome aligns with previous studies that reported a significant link between children’s FMS and academic achievement [9][161718–19]. The present study also revealed that object control had a significant and positive correlation with Chinese and mathematics test scores. Academic performance in mathematics had a significantly positive relationship with stability skills but had no relationship with locomotor skills. Other researchers have offered potential explanations for the substantial effect of object control and stability skills on academic achievement. Stability skills aid children in discovering their environment and constructing strong spatial relationships that can be used to hone their mathematics skills [61]. Fernandes et al. (2016) [62] observed that hand–eye coordination can help improve an individual’s cognitive abilities and processing efficiency. Subsequently, these skills enhance children’s proficiency in reading and writing essays in Chinese as sell as their logical reasoning in mathematics. Although these findings have been reported, the influence of FMS on academic achievement requires additional examination [34].

### Mediation effect of EF on FMS and academic achievement

Piaget’s theory of cognitive development posits that children actively adapt to their environment through self-construction of knowledge, rather than passively assimilating externally imposed information. In this process, physical movements play a crucial role in cognitive development. The current study indicated that EF partially mediates the effect of FMS on academic achievement in Chinese and mathematics domains. Specifically, FMS facilitates the development of higher-order cognitive processes—including analytical reasoning, strategic planning, and evaluative judgment—which subsequently enhance academic performance [5,30,31,32,33]. This evidence lends empirical support to the theoretical proposition that FMS practice provides children opportunities for active exploration and problem-solving, thereby fostering continuous cognitive maturation and, consequently, improved scholastic outcomes. In addition, shared cognitive mechanisms that are involved in FMS and academic achievement may be able to clarify the correlations among FMS, EF, and academic achievement [63]. For instance, assessment tasks related to FMS require children to pay attention (e.g., walking backward on a narrow balance beam with no evident deviation) and use their working memory (e.g., recalling the sequence or steps of striking a stationary ball). Hence, FMS can support the children’s cognitive and learning processes, which are valuable in increasing their performance in school [64]. Moreover, research on functional neuroimaging reveals that the cerebellum and the prefrontal cortex work together to activate and develop motor skills as well as cognitive functions [63,65]. Hence, the brain regions that stimulate FMS and EF offer insights into the indirect influence of FMS on academic achievement through the mediating role of EF.

### Strength and limitations

This research augments the existing literature by presenting findings on the relationship among the FMS, EF, and academic achievement of schoolchildren in China. Notably, the varying degrees of these correlations indicate the different outcomes of children’s academic performance in Chinese and mathematics. Despite the substantial contributions of this work, several limitations should be taken into consideration. First, given that this study used a cross-sectional design, causal inferences could not be made. Such inferences in the relationship between FMS and academic achievement can be determined through longitudinal and interventional studies. Second, parental reports were the basis for measuring EF in this study. Previous studies have confirmed the reliability and validity of this approach. However, the study cannot rule out the subjectivity of parental reports, which may lead to biases in the evaluation of results. Future research can also apply objective measures of EF (e.g., computer-based tasks using E-Prime Software and NIH-Toolbox Cognitive Battery) to enhance the accuracy of the results. Third, this study excluded other potential mediating variables, such as motivation, emotion knowledge, and physical fitness. These factors are also pertinent in understanding the correlation between FMS and academic achievement. Therefore, researchers should consider examining the effects of these mediators. Furthermore, the study incorporated participants spanning a broad age range (6 ~ 12 years), encompassing three distinct stages of cognitive development: the Preoperational Stage, Concrete Operational Stage, and Formal Operational Stage. However, the sample sizes for the Preoperational Stage (N = 14) and Formal Operational Stage (N = 22) were particularly limited. Consequently, these subgroup sizes were insufficient for robust statistical analysis of cognitive function variations across the three developmental stages (categorized as ages 6, 7 ~ 11, and 12 years). Future research should prioritize recruiting larger and more balanced samples across these stages to rigorously examine developmental differences in cognitive trajectories.

## Conclusions and implications

This study examines how FMS and EF predict and influence children’s academic achievement in Chinese and mathematics. Results reveal that object control and inhibition control strongly aid children’s academic performance whereas stability skills and working memory only predict children’s achievement in mathematics and Chinese, respectively. Furthermore, EF mediates the correlation between FMS and academic achievement in Chinese and mathematics.

The findings offer valuable insights into the development of FMS and EF. Parents and teachers in China often focus on children’s academic excellence and neglect the significance of physical activities [66]. Our study outcomes reveal the importance of FMS, which can be developed through kinesthetic engagement. FMS practices, such as climbing, jumping, running, sliding, and playing with balls, have been determined to have an effect on children’s academic achievement. Therefore, parents and teachers should open avenues and motivate their children to participate in physical activities. Effective, interesting, and motivating interventions or programs can promote FMS and EF, which can subsequently foster academic achievement among schoolchildren. Research has confirmed that specialized activities can hone children’s FMS and cognitive abilities. For example, Kashfi et al. (2019) [67] conducted an eight-week intervention program involving balance and coordination exercises and demonstrated that these routines improved the motor skills, working memory, and problem-solving and planning abilities of children with learning disabilities aged 7–9 years. In addition, Magistro et al. (2022) [68] pointed out that two years of physically active lessons, including the development of object control skills and balance skills, enhanced the FMS and EF of first-grade children (6.63 years old). Notably, specific interventions should be implemented to foster children’s performance in Chinese and mathematics. Programs focused on object control (e.g., throwing, catching, and kicking during a ball game) are necessary to boost children’s performance in Chinese and mathematics. Meanwhile, programs emphasizing the development of stability skills (e.g., standing on one leg or balance wood practice) can help strengthen children’s performance in mathematics [61,69]. Given the moderating effect of age, the older the children are, the weaker the influence of FMS on their math scores. Therefore, it is recommended to focus on FMS interventions for lower – grade primary schoolchildren, which can significantly improve their math scores.

## Supporting information

S1 Data (XLSX)
